# Supplementary material for: Mercury Content in Central and Southern Adriatic Sea Sediments in Relation to Seafloor Geochemistry and Sedimentology
Source: Molecules. 2019 Dec 5;24(24):4467. doi: 10.3390/molecules24244467 (PMC6943535; doi:10.3390/molecules24244467)
Supplement: Supplementary file 1 [file molecules-24-04467-s001.pdf]

Supplementary Material

Mercury Content in Adriatic Sea Sediments in Relation to Seafloor Geochemistry and Sedimentology

Elisa Droghini[a], Anna Annibaldi[b,c*], Emanuela Prezioso[b], Mario Tramontana[a,c], Emanuela Frapiccini[d], Rocco De Marco[d], Silvia Illuminati[b,c], Cristina Truzzi[b,c] and Federico Spagnoli[*d]

[a] *Department of Pure and Applied Sciences (DiSPeA), University of Urbino Carlo Bo, Campus Scientifico E. Mattei, 61029 Urbino, Italy*
[b] *Department of Life and Environmental Sciences, Università Politecnica delle Marche, Via Brecce Bianche, 60131 Ancona, Italy*
[c] *CoNISMa, Consorzio Nazionale Interuniversitario per le Scienze del Mare, Piazzale Flaminio, 9, 00196 Roma, Italy*
[d] *Institute of Biological Resources and Marine Biotechnology (IRBIM), National Research Council (CNR), Largo Fiera della Pesca, 2, 60125 Ancona, Italy*
***** Correspondence: federico.spagnoli@cnr.it (F. S.); a.annibaldi@univpm.it (A. A.);

Tab. S1. Mercury content in the sample collected during PERTRE 2016 cruise.

Sample	Hg (mg/Kg)	Longitude	Latitude	Depth (m)	pH	Eh (mV)
1	0.0125	14.782433	43.095	123	7.54	106.7
2	0.0106	14.660867	43.0094	126	7.62	40
3	0.0145	14.535917	43.004467	125	7.56	88.1
4	0.0214	14.4427	42.952233	123	7.51	-105.5
5	0.0444	14.76085	42.864333	256	7.68	229
6	0.0427	14.616117	42.8515	228	7.57	273
7	0.0751	14.302783	42.83455	88.6	7.47	-140.6
8	0.1032	14.195017	42.825033	68.02	7.43	-194
9	0.0247	14.016667	42.80905	14.1	7.38	-233
10	0.0651	14.095467	42.810017	25	7.38	-217
11	0.053	15.611967	42.50605	133	7.55	-98
12	0.0478	15.4435	42.391752	147	7.66	-140.6
13	0.0625	15.281783	42.265283	115	7.52	-23.5
14	0.0581	16.621183	42.204133	176	7.61	-76
15	0.0972	15.116267	42.145617	81	7.52	-177.2
16	0.0712	14.983283	42.111033	40.3	7.36	-200
17	0.0387	14.862283	42.085233	14.8	7.42	-198.5
18	0.0447	16.56225	42.072767	138	7.52	-99
19	0.0668	16.472717	41.9751	108	7.19	-67.2
20	0.0772	16.366717	41.920183	71.7	7.35	-186.1
21	0.0596	16.278183	41.898483	33.3	7.28	-204
22	0.0408	16.216233	41.881017	17.8	7.36	-189
25	0.0348	16.774317	41.712283	131	7.61	141
26	0.0542	16.65685	41.691533	103	7.16	-160.8
28	0.078	16.478217	41.64375	74.4	7.33	-178.5

33	0.0682	16.321533	41.595633	32.5	7.2	-219
35	0.0528	16.09155	41.54635	15.4	7.33	-228
39	0.073	16.550967	41.3771	67.2	7.28	-199.8
43	0.0598	16.762467	41.305533	95.6	5.74	-193
44	0.0393	16.944567	41.299783	114	7.5	37
47	0.0246	17.400217	41.036	112	7.43	-167.5
48	0.0577	17.288433	41.017167	75.4	7.38	-229
50	0.0583	17.979117	40.823717	112	7.64	90
51	0.0669	17.813783	40.76155	76.8	7.59	94.3
52	0.0511	18.153633	40.758633	127	7.57	-81.8
56	0.0806	18.4284	40.530083	104	7.48	89.3
59	0.0814	18.624567	39.90345	113	7.66	169.9
51B	0.0664	17.964783	40.7124	90.9	7.47	95.1
51C	0.0586	18.057867	40.738883	112	7.44	-193.3
54B	0.0806	18.26465	40.548233	88.5	7.32	-209
81B	0.0577	18.291133	40.685383	122	7.51	-126.8
S10	0.0585	15.976233	42.028083	59.9	7.51	-105.5
S11	0.0425	16.091783	42.212967	121	7.68	229
S12	0.0262	15.95785	41.968733	17.9	7.57	273
S13	0.0501	15.863033	42.2806	116	7.47	-140.6
S16	0.0671	15.681933	42.135633	92.7	7.43	-194
S17	0.0635	15.528267	42.020967	52.6	7.38	-217
S18	0.0412	15.94075	42.41265	126	7.55	-98
S19	0.0229	15.45385	41.945217	16.9	7.66	-140.6
S2	0.0171	16.746883	41.9423	171	7.52	-23.5
S20	0.0229	14.282	43.581983	82.7	7.6	136.2
S21	0.0633	14.175067	43.540883	81.4	7.52	-133
S22	0.1139	14.060083	43.498883	67.3	7.47	-154
S23	0.1226	13.95445	43.4603	48.2	7.33	-173.8
S24	0.0863	13.893733	43.43865	29.4	7.29	10.16
S25	0.014	14.388033	43.622333	84.5	7.65	-30
S26	0.021	13.77455	43.386033	13	7.63	-140
S27	0.023	14.4482	43.310833	90	7.68	193.4
S28	0.0567	14.29395	43.25265	78.4	7.5	-163.8
S29	0.0924	14.154367	43.191583	68.9	7.52	-179.6
S3	0.0144	16.796383	42.125	204	7.61	-76
S30	0.0976	14.029733	43.145683	40.5	7.42	-215
S31	0.012	14.600233	43.3555	95.5	7.42	156.3
S32	0.0378	13.914617	43.112717	15.5	7.36	-243
S33	0.0551	15.088583	42.447683	151	7.52	-177.2
S34	0.0674	14.94805	42.398433	128	7.36	-200
S35	0.0826	14.816367	42.348417	95.4	7.42	-198.5
S36	0.0603	14.677717	42.290883	48.6	7.52	-99
S37	0.0491	15.238433	42.496967	154	7.19	-67.2
S38	0.0408	15.309433	42.614067	141	7.35	-186.1
S39	0.0191	14.584217	42.256833	15.2	7.28	-204

S4	0.0447	16.779717	41.465217	109	7.36	-189
S40	0.0431	14.963983	42.74875	207	N.D.	N.D.
S41	0.0459	14.8	42.694583	191	7.61	141
S42	0.0588	14.567883	42.592833	121	7.16	-160.8
S43	0.0918	14.440617	42.539717	85.1	7.33	-178.5
S44	0.0725	14.34675	42.505217	43.1	7.2	-219
S45	0.0445	14.662383	42.632583	148	7.33	-228
S46	0.0382	15.098483	42.83125	183	7.28	-199.8
S47	0.0255	15.005417	42.949583	180	5.74	-193
S48	0.0161	14.279317	42.4779	16.2	7.5	37
S5	0.0567	16.662783	41.42005	90.9	7.13	-191.7
S50	0.0421	17.18855	41.524733	578	7.48	-139.4
S53	0.0632	17.631683	40.875333	86.8	7.34	-186.7
S54	0.0446	17.724533	40.907	108	7.32	-135
S55	0.0546	17.810383	40.9346	119	7.6	181.2
S6	0.0622	16.441533	41.323017	25.7	7.34	-89.5
S61b	0.0467	17.033067	41.951683	609	7.49	-178
S66	0.0467	17.139483	41.176317	122	7.16	-199
S67b	0.0187	17.284383	41.232917	167	7.36	-144.5
S68	0.049	17.007483	41.115317	47.4	7.4	-225
S69B	0.1235	18.523167	39.923367	106	7.56	193.7
S7	0.0306	16.904883	41.509883	132	7.8	105.2
S72	0.0399	18.654567	40.068333	104	7.6	207
S73	0.0402	18.554517	40.067533	94	7.49	-136
S76B	0.0609	18.463617	40.44575	102	7.47	-62.2
S77	0.0493	18.54605	40.44945	121	7.43	-154.8
S78	0.0971	18.389167	40.398533	94.2	7.38	-195.3
S79	0.0699	18.346283	40.544433	102	7.19	-63
S8	0.0533	16.056083	42.156033	115	7.78	97
S80	0.0515	18.52905	40.53465	114	7.45	-113.5
S84	0.0208	18.795917	40.115183	665	7.37	-213
S85	0.0459	18.7127	40.355817	703	7.61	73.5
S86	0.0335	18.614933	40.678683	669	7.53	31.5
S9	0.0778	16.011367	42.09455	99.5	7.49	84.8
S90	0.0476	17.982667	40.944133	138	7.46	-124.8
S92	0.0206	16.976933	41.766417	560	7.47	-142.7
